# Valproic Acid Induces Post-Translational Redox Modifications in Mouse Embryos That Are Prevented via Prior Nrf2 Activation

**DOI:** 10.3390/jdb14030030

**Published:** 2026-07-07

**Authors:** Aubrey Johansen, Kendall Dunford, Garrett Hasegawa, Jason M. Hansen

**Affiliations:** Department of Cell Biology and Physiology, Brigham Young University, Provo, UT 84602, USA

**Keywords:** post-translational modifications, embryo, glutathione, redox potential, Nrf2

## Abstract

Valproic acid (VPA) is a human developmental toxicant that causes neural tube defects and neurobehavioral deficits. Recent work has implicated VPA-induced oxidative stress in cell models of neurodifferentiation, where oxidative post-translational modifications (PTMs) in undifferentiated cells, primarily protein sulfenylation (Pr-SOH), were unique compared to differentiated neurons, primarily protein S-glutathionylation (Pr-SSG). Many of these effects could be mitigated by pretreatments with an Nrf2 inducer. However, it is unclear how early-stage mouse embryos (gestational day 8.5) respond to VPA treatments. Using whole embryo culture, mouse embryos were treated with VPA. A time course assessment of glutathione/glutathione disulfide redox potentials was performed via HPLC throughout 24 h of culture. At 6 h of VPA treatment, embryos were collected for the assessment of protein redox states and specific protein PTMs via various blotting techniques. Also, at 6 h of treatment, the localization of specific PTMs was determined via whole mount staining. Some embryos were pretreated with an Nrf2 inducer. Our data demonstrated that VPA caused a sharp oxidation of redox potentials, which were the greatest between 2 and 6 h, but reverted to control levels by 24 h. Preemptive Nrf2 activation prevented VPA-induced oxidation. Redox blotting showed that VPA caused oxidation of the proteome but this could be reversed by D3T pretreatment. More specifically, Pr-SOH levels increased but Pr-SSG levels were unchanged. Increased Pr-SOH could also be reversed with prior Nrf2 activation. We conclude that embryos at these early stages of development are highly sensitive to VPA and respond more like undifferentiated cells, promoting a more pro-oxidizing outcome for proteins, increasing Pr-SOH formation vs. Pr-SSG. These findings may support specific windows of development where embryos are more susceptible to VPA-induced oxidative injury. Further understanding of redox control and regulation at these susceptible states may serve to develop preventative strategies to reduce poor developmental outcomes after exposures.

## 1. Introduction

Valproic acid (2-propylpentatoic acid; VPA) is a small, branched chain fatty acid that has been used to control seizures in epileptic patients for decades [1]. More recently, VPA has been used for other indications besides epilepsy, including the treatment of bipolar disorder, diabetic neuropathy and migraines [2]. Therapeutically, VPA use has few side effects, yet represents considerable risk to offspring exposed during gestation. Prenatal VPA exposure causes fetal valproate syndrome (FVS), which is characterized by a variety of poor morphological and neurobehavioral outcomes, including but not limited to neural tube defects, facial dysmorphology, attention deficit hyperactivity disorder and autism [3].

Potential teratogenic mechanisms include folic acid antagonism and histone deacetylase inhibition [4,5], but newer evidence suggests VPA-induced oxidative stress may be a key contributing factor as well; it is unknown how it may contribute to the toxic embryonic effects that promote poor developmental outcomes. In embryos, pretreatment with 3H-1,2-dithiole-3-thione (D3T), an Nrf2 inducer of the antioxidant response, greatly reduced the prevalence of VPA-induced neural tube defects, but in Nrf2-knockout embryos, D3T pretreatments were ineffective where NTD rates remained high [6]. These findings suggest a role for VPA-induced oxidative stress as a driver for poor morphological and functional outcomes during development.

More specific to neurogenesis, previous work from our group demonstrates that VPA increases ROS generation in neurogenic cellular models, but that undifferentiated cells were prone to higher levels of ROS generation compared to differentiated cells [7]. Similarly, embryonic GSH/GSSG redox potentials (Eh) were more easily perturbed in undifferentiated cells compared to differentiated cells. Using chemical tags to assess protein redox states, data show that VPA alters protein redox states in undifferentiated cells, being more easily oxidized than proteins in differentiated neurons [8]. Further analyses confirmed that in undifferentiated cells the primary protein post-translational modification was a sulfenic acid (sulfenylation; Pr-SOH), but differentiated cells exhibited an increase in protein *S*-glutathionylation (Pr-SSG) modifications. This unique response between cells is based on their differentiative status, supporting developmental windows where there is likely an increased susceptibility to oxidative toxicants, like VPA, as developmental cell differentiation and fate determinations play out. Interestingly, in vitro pretreatment with D3T prevented cellular oxidative stress and prevented oxidative post-translational modifications to proteins [7,8], suggesting a link to VPA-induced post-translational modifications, especially for Pr-SOH, and aberrant developmental processes.

While VPA-induced oxidative stress and increases in protein post-translational modifications have been well described in in vitro cell models, less is known about the effect of VPA on protein redox states in the intact embryo proper. Here, we address the post-translational effects of VPA on the organogenesis-stage mouse embryo, during the developmental period just prior to neural tube closure, and the potential prevention of NTDs through Nrf2 activation. Understanding the nature of VPA-induced oxidative stress may provide a rationale for how protein targets and pathways are dysregulated during development following oxidant exposures and how these alterations yield dysmorphologies and/or neurobehavioral deficits.

## 2. Materials and Methods

### 2.1. Animals and Whole Embryo Culture

**Figure 1:**
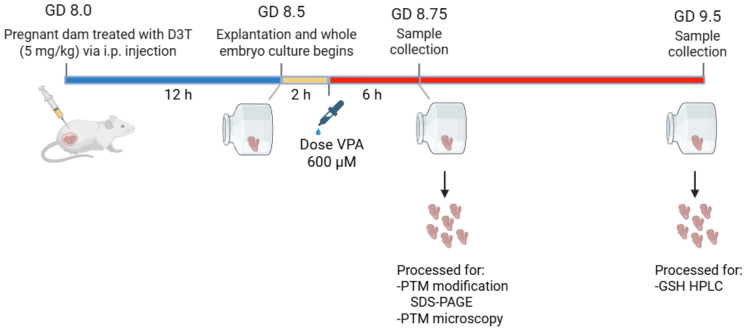
Overview of experimental design in dosing and collection.

CD-1 IGS (Charles River, Raleigh, NC, USA) mice were purchased and relocated to our vivarium facility. Starting at 12 weeks of age, females (n = 45 total) were bred overnight with males of the same strain and approximate age. The following morning, female mice were assessed for evidence of successful copulation through the discovery of a vaginal plug and were then isolated, where this day was designated as gestational day (GD) 0.5 (Figure 1). On GD 8.0, pregnant dams were treated either with 3H-1,2-dithiole-3-thione (D3T; LKT Labs. St. Paul, MN, USA; 5 mg/kg body weight) to induce NRF2 or with vegetable oil for control purposes via intraperitoneal (i.p.) injection. On the morning of GD 8.5, dams were euthanized with CO_2_, and uteri were removed to explant embryos for whole embryo culture as explained previously [6,9]. Previous work has shown that D3T pretreatment was able to robustly increase markers of Nrf2 activation at both the protein and gene expression level in embryos [6]. Embryos were incubated in culture for at least 2 h, after which they were dosed with 600 µM VPA, added directly to the culture medium. This concentration was determined as this is the target therapeutic dose for treating epilepsy (100 µg/mL) [2].

### 2.2. Glutathione Redox Assessment

The glutathione and GSSG concentrations, as well as GSH/GSSG Eh assessments, were measured in intact embryos at the onset of whole embryo culture on GD 8.5 and were collected up to 24 h following initial VPA treatment. In brief, embryos were removed from culture, washed in HBSS, had their extraembryonic membranes removed and then were pooled (6–8 embryos/time point with 4–6 replicate collections at each timepoint) in a 5% PCA solution containing 0.2 M boric acid and 10 µM γ-glutamylglutamate (as an internal standard). At the later stages (8 h and beyond), embryos that did not have active heartbeats were considered non-viable and were not used in the GSH assessments.

Assessments of GSH and GSSG were performed via established HPLC methods [10]. In brief, samples were prepared as *S*-carboxymethylated (to prevent spontaneous oxidation), *N*-dansyl derivatives (for fluorescent detection). Derivatized samples were analyzed using an Arc Separations Module (Waters, Milford, MA, USA) and a Supelcosil LC-NH_2_ 5 µm column (Millipore Sigma, Burlington, MA, USA). Peaks were detected using a 2474 Fluorescence Detector (Waters; excitation 335 nm and emission 518 nm). Redox potentials were calculated using the Nernst equation using intracellular GSH and GSSG concentrations. The midpoint potentials used for redox potential Nernst calculations were −264 mV at pH 7.4 (−240 mV at pH 7.0). Concentrations were normalized based on sample protein levels [10], which were determined via BCA assay methods (Genesee, El Cajon, CA, USA).

### 2.3. Sample Collection for Post-Translation Modification (PTM) Assessment

#### 2.3.1. SDS-PAGE Analysis

Reduced protein redox states were assessed with biotin–iodoacetamide (*N*-[biotinoyl]-*N*′-[iodoacetyl]ethylenediamine; BIAM; Biotium, Fremont, CA, USA), which binds to Pr-SH groups. Embryos were treated as described in Section 2.2. Labeling was performed as described previously [11], but in brief, at collection, embryos were placed in lysis buffer (50 mM Bis-Tris–HCl (pH 6.5), 0.5% Triton X-100, 0.5% deoxycholate, 0.1% SDS, 150 mM NaCl, 1 mM EDTA) containing 50 µM BIAM with protease inhibitors (Genesee, El Cajon, CA, USA). Samples were incubated at 37 °C for 10 min in the dark and then spiked with iodoacetamide (5 mM).

To assess the degree of protein S-glutathionylation (Pr-SSG), on GD 8.5, embryos were pretreated with Biotinylated Glutathione Ethyl Ester (BioGEE) at 250 µM for 2 h prior to co-treatment with 600 µM VPA. Protocols were adapted from previously established methods [12]. At 6 h of VPA treatment (GD 8.75), embryos were removed and placed in ice-cold Hank’s Balanced Salt Solution (HBSS) for the dissection of extraembryonic membranes. Isolated embryos were pooled (6–8 embryos/sample; 4 replicates), placed in RIPA buffer lysis buffer (Millipore Sigma, Burlington, MA, USA) containing protease inhibitors and 5 mM iodoacetamide (IAM; Millipore Sigma), sonicated and incubated at room temperature for 30 min.

For sulfenic acid protein conjugates (Pr-SOH), embryos were also collected on GD 8.75 after 6 h of VPA treatment. Embryos were placed in cold HBSS, and extraembryonic membranes were removed. Isolated embryos were pooled (6–8 embryos/sample; 4 replicates) and collected in RIPA lysis buffer containing protease inhibitors and 3-(2,4-dioxocyclohexyl)propyl 5-((3aR,6S,6aS)-hexahydro-2-oxo-1H-thieno[3,4-d]imidazol-6-yl)pentanoate (DCP-Bio1; 10 µg/mL; Xoder Technologies, Winston-Salem NC, USA), a biotin-tagged detection reagent for protein sulfenic acids [13], sonicated briefly and placed on ice for 1 h.

A BCA protein assay (Genesee, El Cajon, CA, USA) was performed for the quantification of protein in each sample. Gradient (4–20%) SDS-PAGE gels (Bio-Rad, Hercules, CA, USA) were loaded with 20 µg protein/lane in non-reducing conditions and run at 125 V for approximately 70 min. Proteins were blotted onto nitrocellulose membranes (Bio-Rad) using the Trans Turbo Blot system (Bio-Rad) per the manufacturer’s instructions. Membranes were placed in PBS-Tween 20 (PBS-T; 0.1% Tween 20 in PBS) containing a streptavidin–Alexa Fluor 680 conjugate (ThermoFisher, Waltam, MA, USA; 1:5000 dilution) and rocked overnight at 4 °C. The following morning, membranes were washed in PBS-T for 15 min three times at room temperature. Membranes were scanned on an Odyssey CLx infrared scanner (Li-Cor, Lincoln, NE, USA). Quantification of band densitometries was performed using Odyssey Image Studio software (v 6.2).

#### 2.3.2. Embryo Assessment of PTM via Microscopy

After VPA treatments, BioGEE-pretreated embryos were washed in ice-cold HBSS, and the extraembryonic membranes were removed. The embryos were then fixed in 4% paraformaldehyde overnight at 4 °C. The embryos were permeabilized with 0.25% TritonX-100 (Millipore Sigma) in PBS for 1 h at room temperature, after which they were probed with a streptavidin–AlexaFluor 488 conjugate (ThermoFisher, Waltam, MA, USA) for 6 h. The embryos were then washed 3 times with PBS and then stained with 4, 6-diamidino-2-phenylindole (DAPI) for 30 min before additional washes were performed. The embryos were imaged on an FV1000 confocal microscope (Olympus, Needham, MA, USA) as stacked images.

For Pr-SOH conjugates, embryos were removed for culture, and then extraembryonic membranes were removed gently. Embryos were put back into culture in 100% HBSS in the presence of the DCP rhodamine derivative N-(6-(diethylamino)-9-(2-(4-(4-(2,4-dioxocyclohexyl)butanoyl)piperazine-1-carbonyl)phenyl)-3Hxanthen-3-ylidene)-N-ethylethanaminium chloride (DCPRho1; 10 µg/mL; Xoder Technologies, Winston-Salem NC, USA), for 30 min in the dark. After incubation with DCPRho1, embryos were removed, washed 3 times in HBSS and then fixed in 4% paraformaldehyde. The embryos were stained with DAPI for 30 min and then viewed on an FV1000 confocal microscope as stacked images.

The image intensity was determined after images were imported into and analyzed with ImageJ (Bethesda, MD, USA, https://imagej.net/ij/) for both DCP-Rho1 (red) and Streptavidin–AlexaFluor 488 (green) dyes. The intensities were compared to control intensities and are presented as percentages of control intensities (where the control intensities were 100%). As such, values are presented as percent changes from control intensities. Statistical analyses were performed on these values as described below.

### 2.4. Statistics

Statistical comparisons were performed using a one-way analysis of variance (ANOVA), which was then followed by pairwise *t*-tests using the Bonferroni correction. Quantitative data are presented as means ± standard errors of the mean (SEMs). Asterisks denote a statistically significant difference (at least *p* < 0.05).

## 3. Results

### 3.1. Glutathione Redox Potentials Are Oxidized by VPA but Rescued by D3T Pretreatments

**Figure 2:**
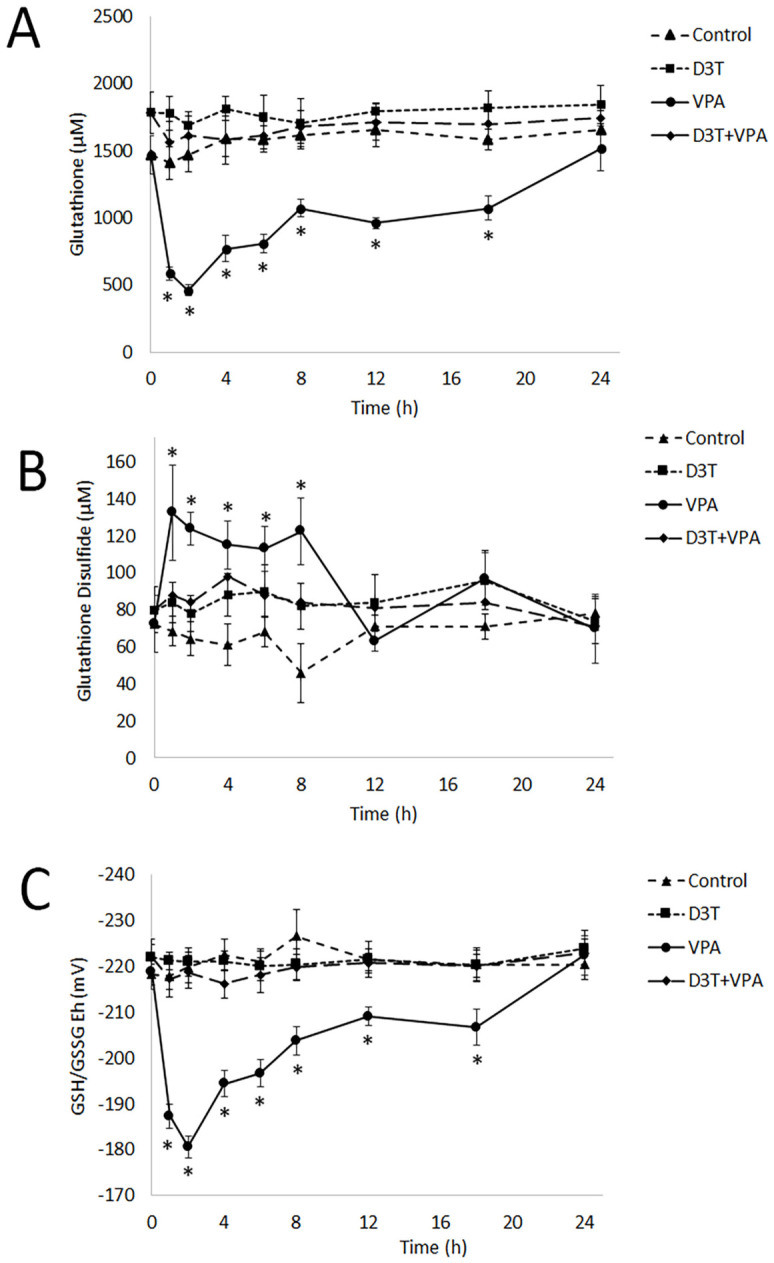
Redox changes in embryos treated with VPA (600 µM), D3T (pretreated 5 mg/kg) or VPA with D3T. (**A**) Glutathione concentrations for up to 24 h following VPA treatment (closed circles). Control (untreated; Closed triangles) and D3T-pretreated (closed squares) embryos are also included. D3T pretreated embryos (12 h prior) were also treated with VPA for up to 24 h (denoted as D3T+VPA; closed diamonds). Glutathione was substantially depleted with VPA compared to control concentrations for 18 h but then returned to control levels. No effect with other treatments or combinations of treatments was observed. (**B**) Glutathione disulfide concentrations are shown as being significantly affected with VPA treatments for the first 8 h of treatment but returned to control levels by 12 h. Other treatments did not affect glutathione disulfide levels. (**C**) Redox potentials (Eh) were significantly affected just in embryos treated with VPA only for 18 h of culture but returned to control values by 24 h. Other treatments did not affect GSH/GSSG Eh. n = 7–8 samples of 3–4 embryos/sample for each time point. Asterisks (*) denote statistically significant differences from control embryo values (*p* < 0.05).

Embryos were treated on GD 8.5 for up to 24 h with 600 µM. Embryos were removed periodically throughout the 24 h window (from GD 8.5 to 9.5). The glutathione concentrations in control, untreated embryos were relatively unaffected during culture (Figure 2A; 1465 ± 143.3 µM at time 0 h), but VPA treatments significantly decreased the concentrations of reduced GSH by nearly 60% (584 ± 49.4 µM) by 1 h and by 69% (460 ± 40.1 µM) by 2 h compared to the GSH levels at time 0 h. The 2 h timepoint constituted the period of the lowest GSH concentrations in VPA-treated embryos, after which the GSH concentrations slowly started to increase back to control levels. Albeit rebounding, GSH levels remained significantly lower than controls until finally reaching control levels at 24 h. Embryos pretreated with D3T did show a 21% increase in GSH (1780 ± 158.2 µM), but it was not statistically significant from that in control, untreated embryos. Embryos that were pretreated with D3T and then treated with VPA did not demonstrate any changes to GSH concentrations throughout the culture.

Glutathione disulfide levels were stable throughout the 24 h culture and did not significantly differ from those in the control, untreated embryos at time 0 h (72 ± 15.2 µM) (Figure 2B). Conversely, VPA treatments caused a significant increase in GSSG of nearly 83% (132 ± 25.8 µM) at 1 h, which remained significantly elevated until 12 h, when GSSG levels returned to normal. In D3T-pretreated embryos, GSSG levels were not significantly different from those in the control, untreated embryos. Similarly, D3T-pretreated embryos that were treated with VPA in culture did not show any statistical differences compared to D3T-pretreated-only embryos.

Glutathione redox potentials in control embryos were also stable through the 24 h culture and did not shift with any significance compared to time 0 h, untreated embryos (−218 ± 3.8 mV) (Figure 2C). VPA treatments caused a significant, robust shift in GSH/GSSG Eh by approximately +31 mV (±2.6) by 1 h and then nearly +38 mV (±2.4) by 2 h, which is the time point of the greatest Eh shift. The redox potentials remained significantly oxidized throughout the rest of the culture, with the exception of the 24 h time point, where Eh had returned to normal control levels (−220 ± 3.3 mV). The embryonic Eh in D3T-pretreated embryos were not statistically different from those in control, untreated embryos. Similarly, the Eh in embryos pretreated with D3T and then treated with VPA were also not significantly different. The significance in GSH/GSSG Eh appears to mostly be a primary consequence of a decrease in GSH availability and then, secondarily, an increase in GSSG concentrations early in the culture (8 h). From the 12 to 18 h time points, GSH/GSSG Eh is a primary function of low GSH levels as GSSG concentrations are mostly re-regulated to normal, control levels.

### 3.2. Embryos Treated with VPA Increase Pr-SOH Formation, but Not Pr-SSG, but This Can Be Prevented by D3T Pretreatment

**Figure 3:**
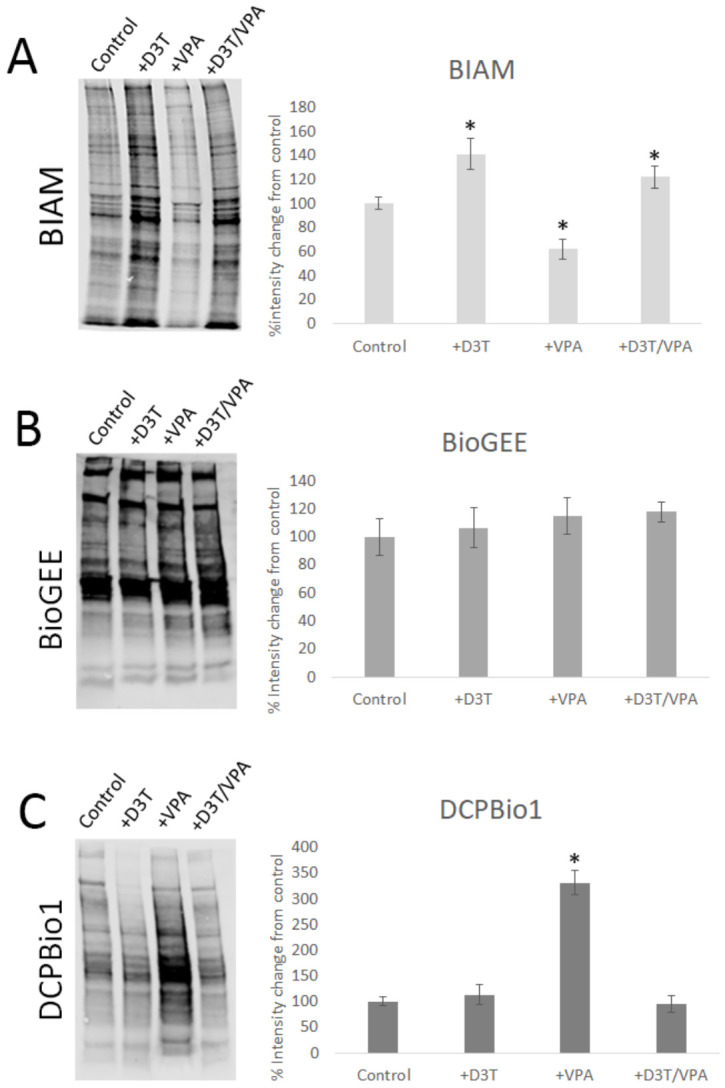
Determination of post-translational modifications following VPA (600 uM), D3T (5 mg/kg) pretreatment, or VPA with D3T pretreatments. Select dams were pretreated with D3T (5 mg/kg) on GD 8.0 before collection for culture on GD 8.5. Once in culture, embryos were treated for 6 h with or without VPA (600 µM). Embryos were collected for analysis of proteins that were reduced (BIAM), or oxidatively modified via either *S*-glutathionylation (Pr-SSG) or sulfenic acid protein (Pr-SOH) formation. (**A**) BIAM blots show bands that represent reduced protein, where darker bands indicate more reduced states. Densitometry of lanes was determined as described. VPA significantly decreased the degree of reduced embryonic proteins, but D3T and VPA with D3T pretreatment caused a significant increase in reduced protein redox states. (**B**) BioGEE blots demonstrate levels of Pr-SSG where bands represent the level of *S*-glutathionylation. No significant quantitative changes in Pr-SSG formation were detected for all treatment groups. (**C**) DCPBio1 blots demonstrate relative levels of Pr-SOH modifications. Treatment with VPA for 6 h caused an increase in embryonic Pr-SOH levels, but these were re-regulated in embryos pretreated with D3T prior to culture. Graphical data is representative of four independently run experiments. Asterisks (*) denote a statistically significant difference from control levels (*p* < 0.05).

Embryos were collected and pooled based on treatments, where each sample represents 6–8 embryos (n = 4–5). Treatments with VPA cause a significant decrease in reduced proteins (Figure 3A). The BIAM labeling of Pr-SH was diminished with VPA treatments, with nearly a 40% decrease based on band intensities. Pretreatment of embryos with D3T prevented VPA-induced protein oxidation and increased the degree of protein reduction from control (nearly 20% more reduced based on band intensity), suggesting that D3T-induced Nrf2 activation prevents VPA-induced protein oxidation.

Embryonic proteins probed with BioGEE for Pr-SSG were determined not to be affected by VPA treatments, where the densitometries of streptavidin-positive bands were like those of control, untreated samples (Figure 3B). Similarly, pretreatment with D3T or with the combination of D3T and VPA also did not show a significant difference in band densitometries, suggesting that D3T also had little effect on embryonic protein *S*-glutathionylation.

Unlike Pr-SSG, Pr-SOH levels were significantly increased with VPA treatments, where Pr-SOH levels were increased nearly 3-fold from those which were observed in the untreated controls (Figure 3C). Interestingly, embryos that were pretreated with D3T did not show any changes to Pr-SOH levels, but those that received the D3T pretreatment and VPA demonstrated significantly decreased levels of Pr-SOH compared to those receiving VPA only. The D3T pretreatment corrections to Pr-SOH levels were statistically identical tobaseline levels in controls.

### 3.3. Whole Mount Embryos Show an Increase in Pr-SOH but Not Pr-SSG Levels

**Figure 4:**
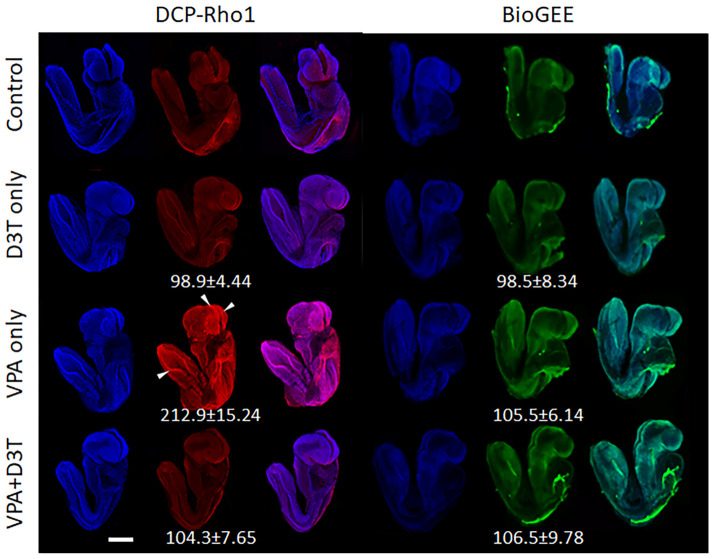
Confocal images of embryos that were exposed to VPA (600 µM) pretreatment, D3T (5 mg/kg) pretreatment, or VPA with D3T pretreatment for assessment of protein post-translational modifications. Valproic acid treatments were performed for 6 h in culture. Embryos were processed and then probed with either DCP-Rho1 (red) for Pr-SOH or BioGEE (subsequently detected with AlexaFluor 488–streptavidin conjugate, green) for Pr-SSG formation. Embryos were also stained with DAPI (blue). In embryos treated with VPA only, an increase in DCP-Rho1 was detected but not in BioGEE-probed embryos, suggesting a preferential production of Pr-SOH versus Pr-SSG. While there was a general increase in Pr-SOH formation throughout, white arrows in VPA-treated embryos denote an even greater Pr-SOH along the edges of the open neural tube and somites. White numbers represent the percent change from control intensity (n = 6–8 embryos; Mean ± SEM) with the similar staining group. Pretreatments with D3T prevented increases in DCP-Rho1 staining.

As a follow up to the SDS-PAGE work, whole mount embryos were used to determine the localization of both Pr-SOH and Pr-SSG formation. The levels of Pr-SSG were unchanged with VPA treatments compared to the control (Figure 4). Neural folds in the cranial region did show an increase in staining but the degree was not increased with VPA. Moreover, D3T pretreatments and D3T pretreatments with VPA treatments did not alter embryonic Pr-SSG. This data correlated with what was observed in SDS-PAGE analyses.

Assessment of Pr-SOH was performed with DCP-Rho1. Here, VPA treatments cause a substantial increase in DCP-Rho1 staining compared to control embryos. Most tissues showed an increase in Pr-SOH staining, but the areas that were most affected were the neural folds and neural crests, in both the cranial and caudal regions. Somites also appeared slightly affected by VPA treatments. Pretreatment with D3T reverted Pr-SOH levels to those observed in the controls.

## 4. Discussion

Numerous teratogenic mechanisms have been proposed to better understand the effects of VPA on the developing embryo, including folic acid antagonism, reactive arene oxide metabolism and inhibition of histone deacetylases (HDACs) [14]. More recently, emerging evidence supports VPA’s induction of oxidative stress. In vitro work showed that, in freshly isolated hepatocytes, VPA induced ROS production, where ROS production was even more exacerbated by the VPA metabolite E-2,4-diene VPA by nearly 300% compared to VPA itself [15]. Interestingly, other studies using hepatocytes also demonstrated an increase in ROS production and showed an increase in 15-F_21_-isoprostane levels, a biological marker of arachidonic acid oxidation, in a dose- and time-dependent fashion [16]. Prior pharmacological depletion of GSH caused hyper-sensitization to VPA treatments (disruption of mitochondrial membrane potentials), suggesting that GSH plays a major role in VPA toxicological responses and the mitigation thereof. Numerous acute myeloid leukemia cell lines receiving VPA treatments showed a statistically significant increase in ROS production and prostaglandin D2 and a concomitant decrease in GSH:GSSG ratios [17]. Similarly, GSH levels were significantly decreased in Jurkat and U937 cells following VPA exposures [18,19]. In animal models treated with VPA, rat brain samples showed an increase in malondialdehyde (MDA), a marker of oxidative stress, and a decrease in a variety of antioxidant enzyme activities [20], suggesting an increase in oxidative damage and a simultaneously lowered ability to mitigate oxidants. Additionally, acute VPA treatments in rats caused a rapid depletion of GSH in hepatic samples, but these effects were exacerbated within the mitochondria, but in chronic dosing regimens, these effects were not observed [21]. Conversely, other studies using chronic VPA administration showed an increase in MDA levels in both the serum and testes, which also coincided with lowered activity levels of the antioxidant enzymes glutathione peroxidase and superoxide dismutase [22]. Many of these effects could be reversed with antioxidant supplementation. Interestingly, reactive metabolites of VPA, specifically (*E*)-2-propyl-2,4-pentadienoic acid, were shown to conjugate to GSH. These studies, and others, appear to support the rationale that VPA may exert toxicity primarily through oxidative stress. Unfortunately, less is known about the role of VPA-induced oxidative stress in the embryo or how it translates to alterations to developmental processes required for conventional embryogenesis.

Here, we demonstrate that cellular redox states are highly dysregulated in murine embryos treated with VPA in culture. The disruption of cellular GSH/GSSG Eh is temporary, resolved after 24 h, which demonstrates that the embryo has the capability to inherently re-regulate redox states independently of maternal involvement. However, the length of VPA-induced disruption may serve to disturb redox-sensitive processes that are developmental stage-specific, where delays in essential developmental events may contribute to the perturbation of normal embryogenesis. Furthermore, VPA-induced GSH/GSSG Eh oxidation in embryos positively correlated with levels of superoxide dismutase 2 (SOD2) acetylation [23], where acetylated SOD2 increases ROS production [24,25]. However, how VPA-induced shifts in GSH/GSSG Eh occur in the embryo is not well understood and is critical for developing strategies to prevent oxidative damage and to promote more protective endpoints to decrease poor developmental outcomes.

The present work correlates well with previous studies, where, using the P19 model of neurogenesis [26], the GSH/GSSG Eh in undifferentiated P19 cells, when treated with VPA, were significantly oxidized (approximately +15 mV), but in P19-derived differentiated neurons, GSH/GSSG Eh was unaffected and remained consistent with control, untreated neurons [7,8], suggesting that undifferentiated cells are more prone to VPA-induced redox disruption. When VPA was administered during P19 cellular differentiation, cells in earlier differentiation periods were more prone to not fully differentiate, whereas cells treated during more late-stage differentiation periods differentiated more normally [7].

In this embryo model, GD 8.5-staged embryos have undergone the formation of various germ layers but are still in the early stages of development, where very little terminal differentiation has occurred. In particular, the development of the neural plate, neural groove and neural folds, and the migration of neural crest cells contribute to a myriad of key morphological features and developmental events that culminate in the closure of the neural tube that occur from GD 8.5 to GD 9.5. The GSH/GSSG Eh was dysregulated by VPA treatments, and the magnitudes of the GSH/GSSG Eh shift were significant, comparable to those observed in undifferentiated P19 cells. These data suggest that during this critical period of embryonic neural tube development (GD 8.5–9.5), VPA affects the embryo as if it were more of an undifferentiated cellular phenotype. The basic tenets of developmental toxicology describe periods of development where the embryo is vulnerable to toxicant exposures [27], where differential oxidative susceptibilities may be due to differences in cellular biochemical, structural and metabolic stage-specific differences. The exact characteristics that allow for earlier stages to be more susceptible to VPA-induced oxidative damage remain unknown.

In both in vivo and in vitro studies, findings show that D3T pretreatments prevented VPA-induced GSH/GSSG Eh oxidation, stabilizing redox states and leading them to remain unchanged, even in undifferentiated cells, and decreased the rates of NTDs [6,7]. Pretreatment with D3T in utero has been shown to upregulate many positive markers of Nrf2 activation, likely contributing to embryonic protection. Interestingly, protective effects of D3T against VPA-induced NTDs were not observed in Nrf2-knockout mice [6], purporting Nrf2 as a key pathway in embryonic protection from oxidative stress. Our data here support the idea that many of the beneficial effects observed with preserving differentiation and promoting neural tube closure appear to be mediated through Nrf2 by establishing and preserving cellular redox environments.

The dysregulation of protein redox states is supported by BIAM blots (Figure 3A), where VPA caused an overall decrease in the amount of reduced embryonic proteins. However, BIAM blots do not fully show the type of oxidative modification present but rather simply show that there is a shift in available Pr-SH levels. Similar findings were described previously in cells [8].

**Figure 5:**
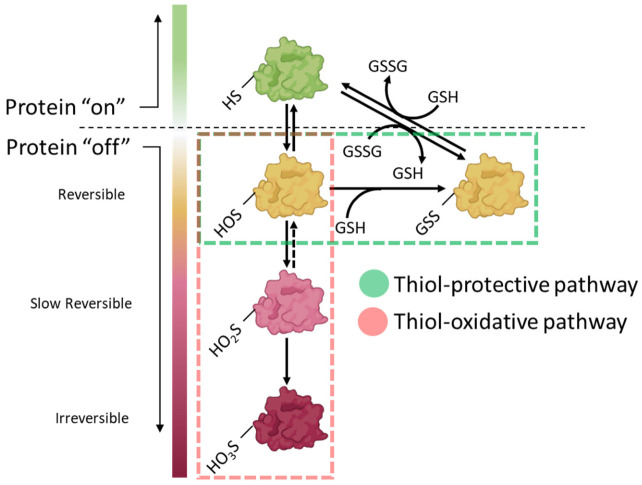
Oxidative modifications of proteins. Sulfenylation of proteins can initiate hyperoxidation of proteins to a sulfinic acid (Pr-SO_2_H) or sulfonic acid (Pr-SO_3_H), leading to slow recovery or permanent oxidized states (thiol-oxidative pathway). Conversely, Pr-SOH can be modified via *S*-glutathionylation to Pr-SSG, which protects the protein from further oxidative damage (thiol-protective pathway).

A key difference from previous work is that undifferentiated cells exposed to VPA showed increased protein sulfenylation, Pr-SOH, but no upregulation of Pr-SSG formation [8], yet in P19-derived neurons (differentiated), VPA promoted *S*-glutathionylation but with very little sulfenylation. Pr-SOH can further oxidize to sulfinic acids (sulfinylation; Pr-SO_2_H) and then to sulfonic acids (sulfonylation; Pr-SO_3_H). Pr-SO_2_H is reversible, albeit reduction is slow, but unlike Pr-SO_2_H, Pr-SO_3_H is irreversible and will often designate the protein to be degraded [28]. Alternatively, the formation of Pr-SSG is suggested to protect proteins from over-oxidation during periods of oxidative stress by preventing hyperoxidation [29]. Pr-SOH can be *S*-glutathionylated to Pr-SSG, where Pr-SSG can be reduced back to Pr-SH when GSH/GSSG Eh favors a more reducing environment [30]. As such, cellular redox potentials may dictate cysteine switch states in proteins to control protein function during development [31], where naturally occurring redox shifts control important developmental events, but aberrant switching due to chemical-induced oxidative stress in the embryo could have serious effects where processes could be diminished or altogether inhibited. In embryos, Pr-SSG formation following VPA exposures was low, but Pr-SOH levels were dramatically increased, which again reinforces more undifferentiated-type cellular responses at this stage of development. These results support the period of early neurulation as a sensitive developmental phase to oxidative insults like VPA, allowing protein sulfenylation but not *S*-glutathionylation and promoting a more thiol-pro-oxidizing pathway versus a more thiol-protective one and, therefore, support a more damaging outcome (Figure 5). Although neither Pr-SO_2_H nor Pr-SO_3_H was measured here, the finding that Pr-SOH did increase without a concomitant increase in Pr-SSG suggests that proteins are shunted away from *S*-glutathionylation protection and are more likely to become hyperoxidized, and thus, protein function is more perturbed or perhaps permanently dysregulated.

Our data here purport that GD 8.5 embryos are more “undifferentiated” in nature, behaving like undifferentiated cells, and likely capture a period of greater susceptibility to chemical insults like VPA. These unique differences in PTM preferential outcomes may rationalize specific periods of greater toxicant susceptibility. Proteins that form either Pr-SO_2_H, which are slow to be reduced, or Pr-SO_3_H, which are prone to degradation, are likely to have much more severe consequences in a developing system. Proteins that form Pr-SSG are not as likely to disrupt signaling and cellular function for as long, comparatively. Interestingly, the prevention of Pr-SOH formation with D3T pretreatment likely provides evidence for protection from NTD formation as observed [6].

## 5. Conclusions

In conclusion, embryos treated with VPA show the hallmarks of oxidative stress, including GSH/GSSG Eh shifts and the formation of oxidative PTMs. Furthermore, activation of the Nrf2 systems prevents these changes. Together, these data further support the idea that VPA-induced embryotoxicity occurs through an oxidative mechanism. During development, as cellular shifts in metabolic and antioxidant capacity are adapting, early stages appear to be more prone to redox disruption and may narrow the periods of greatest susceptibility to VPA. Clearly, more work is required to further identify protein targets of VPA-induced oxidative stress and how they regulate processes that are related to the observable developmental outcomes.

## Data Availability

Data is contained within the article.

## Abbreviations

The following abbreviations are used in this manuscript:

BIAM	Biotinylated iodoacetamide
BioGEE	Biotinylated Glutathione Ethyl Ester
D3T	3H-1,2-dithiole-3-thione
DAPI	4, 6-diamidino-2-phenylindole
DCP-Bio1	3-(2,4-dioxocyclohexyl)propyl-5-((3aR,6S,6aS)-hexahydro-2-oxo-1H-thieno[3,4-d]imidazol-6-yl)pentanoate
Dcp-Rho1	N-(6-(diethylamino)-9-(2-(4-(4-(2,4-dioxocyclohexyl)butanoyl)piperazine-1-carbonyl)phenyl)-3Hxanthen-3-ylidene)-N-ethylethanaminium chloride
Eh	Redox potential
FVS	Fetal Valproate Syndrome
GD	Gestational Day
GSH	Glutathione
GSSG	Glutathione disulfide
HBSS	Hanks Balanced Salt Solution
IAM	Iodoacetamide
MDA	Malondialdehyde
Nrf2	Nuclear Factor Erythroid 2-Related Factor 2
NTD	Neural Tube Defect
Pr-SH	Protein Thiol
Pr-SOH	Protein sulfenic acid
Pr-SO_2_H	Protein sulfinic acid
Pr-SO_3_H	Protein sulfonic acid
Pr-SSG	*S*-glutathionylated Protein
ROS	Reactive Oxygen Species
VPA	Valproic acid
